# Paediatric gastroesophageal reflux disease and parental mental health: Prevalence and predictors

**DOI:** 10.1177/13591045231164866

**Published:** 2023-03-20

**Authors:** Elizabeth GM Aizlewood, Fergal W Jones, Rachel M Whatmough

**Affiliations:** Salomons Institute for Applied Psychology, 2238 Canterbury Christ Church University, Tunbridge Wells, UK

**Keywords:** infant reflux, parent mental health, infant gastroesophageal reflux disease, gastroesophageal reflux disease, GERD, GORD, well-being

## Abstract

**Objective:**

The current study aimed to estimate the prevalence of common mental health difficulties in parents who have an infant with Gastroesophageal Reflux Disease (GORD), and to identify psychological predictors of parental anxiety, depression, and well-being, as a platform for subsequent intervention development.

**Methods:**

Parents of infants with GORD (*N* = 309) completed online psychometric measures of potential predictors (self-compassion, illness appraisals, and illness uncertainty), potential confounders (sleep quality, relationship satisfaction, social support, and infant feeding satisfaction), and mental health outcomes (anxiety, depression, and wellbeing). The outcome measures were repeated eight-weeks later (*N* = 103).

**Results:**

At the first time-point, 66% of participants exceed the clinical cut off for generalised anxiety disorder and 63% exceeded that for a depressive disorder. Both had significantly reduced eights-weeks later. Greater self-compassion predicted lower anxiety and depression, and better well-being, in both cross-sectional and longitudinal data, including when all confounders were controlled for. Illness uncertainty and illness appraisals were less consistent predictors. No robust differences were found between parents of infants with silent GORD and those with GORD with visible regurgitation.

**Conclusions:**

Parents of infants with GORD showed high rates of anxiety and depression, which were elevated compared to those that have been found in perinatal and general population samples. Self-compassion was a consistent predictor of better mental health and has promise as a proximal intervention target. Future research could benefit from examining the efficacy of a compassion-focussed intervention in this population.

## Introduction

Infant gastroesophageal reflux disease (GORD) may be diagnosed “when symptoms of reflux become severe and need medical treatment” (National Institute for Health and Care Excellence, 2016). Common characteristics of GORD in infants include effortless regurgitation of feeds, displays of pain or marked distress, sleep disturbances and persistent crying (Blanch & Reflux Infants Support Association, 2010). These symptoms have the potential to negatively impact parents' mental health. For example, persistent infant crying, or infantile colic, has been linked to maternal depression (Petzoldt, 2018). In addition, higher levels of parenting stress (Miller-Loncar et al., 2004), sleep disturbance and feeding difficulties are associated with maternal depression and anxiety (Chaput et al., 2016; Meltzer & Moore, 2008). Moreover, infant GORD occurs during a period of particular vulnerability to the onset and relapse of parental mental health problems (Aktar et al., 2019).

The one study to date that has examined the prevalence of mental health problems in parents of infants with GORD (in Australia) found that 29% of respondents self-reported a diagnosis of postnatal depression or anxiety, which was significantly higher than the general population rate (Reflux Infants Support Association, 2017). While valuable, this study was limited by not using validated measures of mental health and not being peer reviewed. In addition, alongside prevalence, it would be helpful to understand the predictors of parental mental health and wellbeing in parents of infants with GORD, not least because this could identify proximal targets for interventions aimed at supporting these parents.

Although predictors of mental health and wellbeing have not yet been examined in this population, theory and evidence from other childhood illnesses is potentially relevant. In particular, Leventhal et al.’s (2003) model of illness representation proposes five key components of illness cognition that guide illness appraisals, namely identity, timeline, consequence, control-cure and cause, and more threatening appraisals of illness have been consistently associated with poorer parental adjustment in a range of adult and childhood illnesses (e.g. Broadbent et al., 2015). In addition, illness uncertainty, which is not explicitly included in Leventhal et al.'s model, is a particular appraisal that might be relevant to infant GORD, because of the difficulties diagnosing this condition and associated uncertainty (Gonzalez-Ayerbe et al., 2019). In other childhood illnesses, such uncertainty is associated with higher parental distress, raising the possibility that the same may be true for parents with infants with GORD (Wright et al., 2009).

Another theoretical construct that has been drawn upon extensively in the literature on parental well-being is self-compassion (e.g. Felder et al., 2016). Neff (2003) argues that self-compassion comprises three aspects: being kind towards oneself when facing failure or suffering; recognising such experiences as part of wider human experience; and being present with associated thoughts and feelings without over-identifying with them (Neff, 2003). Self-compassion is thought to aid mental health by reducing self-criticism and rumination. Consistent with this, self-compassion is associated with higher levels of parental well-being in the postnatal period (Felder et al., 2016). However, no research to date has explored the role of parental self-compassion in the context of infant GORD.

Additional factors that have been linked with parental mental health that are likely to be relevant include sleep (Meltzer & Moore, 2008), partner relationship satisfaction (Rosand et al., 2011), perceived social support (Tak & McCubbin, 2002) and satisfaction with the infant’s feeding (Hall & Hauck, 2007).

In summary, the prevalence of parental mental health difficulties in the context of infant GORD has thus far only been examined using a non-validated questionnaire and, while there is theory and evidence from other childhood conditions regarding predictors of parental wellbeing, these have yet to be examined for infant GORD. Therefore, the current study aimed to estimate the prevalence of mental health difficulties in the population of parents who have an infant with GORD using validated measures, and to test predictors of parental anxiety, depression and well-being in this population. It was hypothesised that more threatening appraisals of the infant’s GORD and higher illness uncertainty would predict higher anxiety and depression and lower well-being scores, in both cross-sectional data and at an eight-week follow-up. It was also hypothesised that higher parental self-compassion would predict lower anxiety and depression and higher well-being scores. The study also aimed to explore differences between standard GORD and a type of GORD known as silent reflux; the latter being when the contents of the stomach move up the oesophagus, but do not enter the mouth, resulting in no visible regurgitation (National Institute for Health and Care Excellence, 2015). Anecdotally, it takes longer to diagnose GORD when the reflux is silent and parents experience greater uncertainty (Blanch & Reflux Infants Support Association, 2010). Therefore, it was tentatively hypothesised that parents with infants with silent reflux would show greater illness uncertainty and poorer depression, anxiety and wellbeing than those with infants with standard GORD.

## Methods

### Design

The study employed a cross-sectional, online, survey comprising a series of self-report outcome measures, as well as demographic and GORD-related questions. A subset of measures was repeated after eight-weeks, again by online survey.

### Participants and recruitment

Participants were recruited via UK-based Facebook groups for parents of infants with reflux. Participants were eligible if they identified as a parent or primary caregiver of an infant (aged 3–12 months) who had been diagnosed with GORD by a prescribing physician (e.g. paediatrician or general practitioner) and who was currently receiving treatment for GORD. Field (2013) suggested a sample size of 119 participants would be sufficient to detect a medium effect size, for a conventional level of power (.80) and up to 10 predictors. Details of the recruited sample and participant flow through the study are provided in the results. Ethical approval was granted by a university research ethics committee and all participants provided informed consent.

### Measures

All the following measures were administered at the first time-point using Qualtrics survey software. The measures of parental anxiety, depression and well-being, together with those listed in the control variables section below, were repeated eight-weeks later.

#### Outcome variables

Parental anxiety was measured using the *Generalised Anxiety Disorder 7* (GAD-7), a widely used, self-report, screening measure of generalised anxiety, validated in large clinical and general population samples (Löwe et al., 2008; Spitzer et al., 2006). In the current study, internal consistency was excellent (*α* = .91).

Parental symptoms of depression were measured using the *Patient Health Questionnaire 8* (PHQ-8), a self-report scale that has demonstrated good reliability and validity in clinical and general population samples (Kroenke et al., 2009). Internal consistency in the current sample was also good (*α* = .88). The PHQ-8 was employed instead of the PHQ-9 for ethical reasons. Specifically, the PHQ-9 (unlike the PHQ-8) asks about suicidal and self-harm ideation, but we did not need to ask participants this potentially distressing question, as the PHQ-8 has good psychometric properties.

Parental well-being was measured using the self-report *Short Warwick-Edinburgh Mental Well-Being Scale* (SWEMWBS), which has good psychometric properties (Haver et al., 2015), along with a good internal consistency in the current study (*α* = .86).

#### Predictor variables

Participants' perceptions of uncertainty in the context of having an infant with GORD was measured using the *Parent Perception of Uncertainty Scale* (PPUS; Mishel, 1983). The wording of two items was altered to be more applicable to this study’s population: “It is vague to me how I will manage the care of my child after he/she leaves hospital” was changed to “It is vague to me how I will manage the care of my child”, and “I can depend on the nurses to be there when I need them” was changed to “I can depend on health professionals to be there when I need them”. Internal consistency was found to be excellent (*α* = .92).

Participants' illness appraisals were measured using the *Brief Illness Perception Questionnaire* (B-IPQ, Broadbent et al., 2006), which has good psychometric properties (Broadbent et al., 2015), and a good internal consistency in the current sample (*α* = .80).

Participants' self-compassion was measured using the *Self-Compassion Scale -Short Form* (SCS-SF; Raes et al., 2011), which has demonstrated near perfect correlation with the valid and reliable long form SCS (r ≥ .97). Internal consistency in the current sample was good (*α* = .80).

#### Control variables

All control variables were assayed using single item measures, to reduce the length of the survey and hence participant burden (Hoeppner et al., 2011). All these measures had existing data indicating adequate psychometric properties. Sleep was measured using the *Sleep Quality Scale* (SQS; Cappelleri et al., 2009); social support by the *Brief Measure of Social Support* (BMSS; Atroszko et al., 2015); and support from personal relationships by the *Scale of Satisfaction with Personal Relationships* (SSPR; Atroszko et al., 2015). In addition, a single item from the *Maternal Breastfeeding Evaluation Scale* (MBES) that measured overall satisfaction with breastfeeding was selected, due to its high correlation with the full scale (r = .83, *p* < .001; Leff et al., 1994). This item was adapted to account for experiences of parents who may not have been breastfeeding their infants. The resulting item read: “In the last 2 weeks, overall, how satisfied have you been with your baby’s feeding? *0 (Very dissatisfied) – 10 (very satisfied*).”

#### Demographic and illness context questions

Participants also provided relevant demographic information and information about their infant’s GORD diagnosis, as detailed in the results section.

### Data analysis

Data were analysed using IBM SPSS version 24. Little’s (1988) Missing Completely at Random (MCAR) test examined the nature of the missing data. Assumptions of the employed tests were checked and sufficiently met. Descriptive statistics were used for prevalence data, and simple and multiple regressions employed to explore the relationships between predictor (and control) variables and outcomes. Paired sample *t*-tests examined whether there was a difference between standard GORD and silent-reflux.

## Results

### Sample characteristics

Four hundred and forty-six participants were assessed for eligibility and 74 did not meet the inclusion criteria, the reasons being either that the infant had not been given reflux medication in the past 2 weeks (*n* = 69) or an absence of a diagnosis by a prescribing physician (*n* = 5). This resulted in 372 complete and partial responses for analysis; for further details, see online supplementary material Figure OS-1. Little’s (1988) MCAR test was not significant (χ^2^ = 1932.801, df = 2070, *p* = .985), so the data were treated as MCAR and listwise deletion employed, resulting in a final sample of 309 at baseline and 103 at timepoint 2. There were no significant baseline differences between participants who only completed the initial survey and those who also completed the follow-up (all *p* > .05).

The sample was nearly entirely female and had little representation from Black, Asian and Minority Ethnic (BAME) groups. The mean age of onset of reflux symptoms was 1.24 months (SD = .63, range = 1–6 months) and mean age of receiving a diagnosis of GORD 2.22 months (SD = 1.54, range = 1–12 months). For more details, see Table 1.

**Table 1.** Demographic and infant reflux data (*N* = 309).

Characteristic	Mean (SD) or *n* (%)
Participant’s age (years)	31.72 (4.75)
Number of children	1.76 (0.85)
Age of infant with GORD (months)	6.86 (2.90)
Age reflux symptoms started (months)	1.24 (0.63)
Age of GORD diagnosis (months)	2.22 (1.54)
Time to diagnosis (months)	0.98 (1.34)
Participant gender	
Female	307 (99.4%)
Infant gender	
Male	169 (54.7%)
Female	140 (45.3%)
Country	
United Kingdom	263 (85.1%)
US and Canada	28 (9.1%)
Other	18 (5.8%)
Ethnicity	
White british	250 (80.9%)
White other	48 (15.5%)
Mixed	5 (1.6%)
Black or black british	2 (0.6%)
Other	4 (1.3%)
Employment status	
Full time	136 (44.0%)
Part time	99 (32.0%)
Homemaker	44 (14.2%)
Unemployed/unable to work	14 (4.6%)
Student	8 (2.6%)
Other	8 (2.6%)
Education	
Undergraduate degree	106 (34.3%)
No degree	102 (33.0%)
Postgraduate degree	54 (17.5%)
Professional degree	47 (15.2%)
Marital status	
Married	195 (63.1%)
Co-habiting	88 (28.5%)
Single	18 (5.8%)
Divorced/Separated	3 (0.9%)
Other	5 (1.6%)
Type of infant GORD	
Reflux with regurgitation	142 (46.0%)
Silent reflux	147 (47.6%)
Other	20 (6.5%)
Diagnosing professional	
General practitioner (GP)	157 (50.8%)
Paediatrician	102 (33.0%)
Health visitor	29 (9.4%)
Specialist nurse	8 (2.6%)
Other	13 (4.2%)
Any other diagnoses related to GORD	
Yes	246 (79.6%)
No	63 (20.4%)
Any other physical or developmental diagnoses	
No	268 (86.7%)
Yes	41 (13.3%)

### Estimated prevalence of mental health problems

In the initial survey, 204 of the 309 participants (66.0%; 95% CI: 60.4%–71.3%) scored above the GAD-7 clinical cut off for generalised anxiety disorder (≥10; Spitzer et al., 2006), and 196 (63.4%; 95% CI: 57.8%–68.8%) scored above the cut off for a depressive disorder on the PHQ-8 (≥10; Kroenke et al., 2009). By the 8-week follow-up, these had reduced to 41/103 (39.8%; 95% CI 30.3%–49.9%) and 50/103 (48.5%; 95% CI: 38.6%–58.6%), respectively, with levels of anxiety and depression having significantly improved (respectively *t* (102) = 6.88 and 4.46, *p* < .001). It worth noting that well-being, feeding and sleep satisfaction, and parents’ perceptions of how well their infant’s GORD was managed, all also significantly improved between Time 1 and Time 2 (*p* < .05). For further details, see online supplementary Tables OS-1 and OS-2 and Figures OS-2 and OS-3.

### Cross-sectional predictors of mental health and well-being

In initial, separate, simple linear regressions for each predictor and outcome combination, illness appraisals, illness uncertainty and self-compassion (measured at Time 1) all significantly predicted anxiety, depression and well-being scores, in the expected directions, both when the outcomes were taken from Time 1 and from Time 2 (*p* < .001). See online supplementary Table OS-3 for further details.

Multiple linear regressions were conducted to test the hypotheses that, in the Time 1 cross-sectional data, parents' appraisals of their infant’s GORD, illness uncertainty and self-compassion would predict parental anxiety, depression and well-being, above and beyond the control variables of satisfaction with the infant’s feeding, sleep quality, relationship satisfaction and social support. Separate regressions were run for each of the nine predictor-outcome combinations, with the control variables entered into the first block (Model 1) and the respective predictor added to the second block (Models 2a, 2b and 2c). As indicated in Table 2 by the significant F change from Model 1 to the respective Model 2s, Time 1 illness appraisals, illness uncertainty and self-compassion all significantly predicted Time 1 anxiety, depression and well-being, above and beyond the Time 1 control variables, in the expected directions. The analysis was repeated, but with the three predictors included in the second block of the same regression rather than separate ones, resulting in three regressions (one for each outcome). As can be seen from online supplementary Table OS-4, Time 1 self-compassion and illness uncertainty remained significant predictors of Time 1 anxiety, depression and well-being scores, over and above the control variables. However, illness appraisals were no longer a significant predictor of any of the outcomes.

**Table 2.** Multiple linear regressions at baseline (see main text for details).

Outcome variable	Predictors	Unstand-ardized B	*t* (df)	*R* ^2^	F (df)	Change from the respective model 1	
						*R*^2^ change	F (df) change
GAD-7 Model 1	Social support	−.041	−.294 (304)	.223	21.785*** (4, 304)		
	Relationship	−.601	−4.062*** (304)				
	Sleep	.368	2.554* (304)				
	Feeding	−.605	−5.386*** (304)				
GAD-7 Model 2a	Social support	.025	.183 (303)	.260	21.305*** (5, 303)	**.037**	15.288*** (1, 303)
	Relationship	−.514	−3.513*** (303)				
	Sleep	.298	2.096* (303)				
	Feeding	−.404	−3.329*** (303)				
	Appraisals	.117	3.910*** (303)				
GAD-7 Model 2b	Social support	.043	.314 (303)	.288	24.570*** (5, 303)	**.066**	27.975*** (1, 303)
	Relationship	−.533	−3.743*** (303)				
	Sleep	.318	2.299* (303)				
	Feeding	−.411	−3.611*** (303)				
	Uncertainty	.092	5.289*** (303)				
GAD-7 Model 2c	Social support	.030	.223 (303)	.316	28.049*** (5, 303)	**.094**	41.497*** (1, 303)
	Relationship	−.400	−2.807** (303)				
	Sleep	.331	2.441* (303)				
	Feeding	−.578	−5.469*** (303)				
	Self-compassion	−2.708	−6.422*** (303)				
Well-beingModel 1	Social support	.086	1.044 (304)	.273	28.472*** (4, 304)		
	Relationship	.503	5.837*** (304)				
	Sleep	−.272	−3.243** (304)				
	Feeding	.226	3.459** (304)				
Well-being Model 2a	Social support	.036	.455 (303)	.329	29.737***(5, 303)	**.057**	25.586*** (1, 303)
	Relationship	.438	5.228*** (303)				
	Sleep	−.220	−2.703** (303)				
	Feeding	.077	1.108 (303)				
	Appraisals	−.087	−5.058*** (303)				
Well-being Model 2b	Social support	.024	.317 (303)	.369	35.395*** (5, 303)	**.096**	46.166*** (1, 303)
	Relationship	.453	5.616*** (303)				
	Sleep	−.236	−3.006** (303)				
	Feeding	.085	1.315 (303)				
	Uncertainty	−.067	−6.795*** (303)				
Well-being Model 2c	Social support	.038	.505 (303)	.389	38.503*** (5, 303)	**.116**	57.474*** (1, 303)
	Relationship	.368	4.540*** (303)				
	Sleep	−.247	−3.205** (303)				
	Feeding	.208	3.459** (303)				
	Self-compass	1.813	7.581*** (303)				
PHQ-8 Model 1	Social support	−.121	−.849 (304)	.244	24.580*** (4, 304)		
	Relationship	−.681	−4.535*** (304)				
	Sleep	.638	4.362*** (304)				
	Feeding	−.400	−3.509** (304)				
PHQ-8 Model 2a	Social support	−.066	−.464 (303)	.269	22.272*** (5, 303)	**.024**	10.096** (1, 303)
	Relationship	−.609	−4.065*** (303)				
	Sleep	.579	3.988*** (303)				
	Feeding	−.233	−1.875 (303)				
	Appraisals	.097	3.177** (303)				
PHQ-8 Model 2b	Social support	−.048	−.344 (303)	.292	24.941*** (5, 303)	**.047**	20.180*** (1, 303)
	Relationship	−.622	−4.251*** (303)				
	Sleep	.594	4.182*** (303)				
	Feeding	−.231	−1.974* (303)				
	Uncertainty	.080	4.492*** (303)				
PHQ-8 Model 2c	Social support	−.038	−.289 (303)	.366	35.023** (5, 303)	**.122**	58.273*** (1, 303)
	Relationship	−.445	−3.151** (303)				
	Sleep	.594	4.425*** (303)				
	Feeding	−.368	−3.516** (303)				
	Self-compass	−3.179	−7.634*** (303)				

***p < .001; **p < .01; *p < .05; GAD-7 = Generalized Anxiety Disorder Screener 7; PHQ-8 = Patient Health Questionnaire 8; Relationship = Relationship Satisfaction; Sleep = Sleep Quality; Feeding = Feeding Satisfaction; Self-Compass. = Self-Compassion; Appraisals = Illness Appraisals; Uncertainty = Illness Uncertainty.

### Longitudinal predictors of mental health and well-being

To examine whether the hypothesised relationships existed in the longitudinal data, the multiple regressions described above for the cross-sectional (Time 1) data were repeated, with the respective outcome variables (i.e. anxiety, depression and well-being) now taken from Time 2 rather than Time 1. All the predictors and control variables remained measured at Time 1. The findings mirrored those in the cross-sectional data, with Time 1 illness appraisals, experience of uncertainty and self-compassion all significantly predicting Time 2 anxiety, depression and well-being, above and beyond the Time 1 control variables, in the expected directions (see Table OS-5 in the online supplementary material, for details).

As with the cross-sectional data, additional regressions were run in which all three predictors were added to the second block of the model together, resulting in three further regressions, one for each outcome (see online supplementary Table OS-6). Mirroring the cross-sectional findings, Time 1 self-compassion was a significant predictor of lower anxiety and depression and higher well-being at Time 2, over and above the Time 1 control variables. However, in contrast to the cross-sectional findings, Time 1 illness uncertainty did not significantly predict any Time 2 outcomes, while Time 1 illness appraisals significantly predicted poorer Time 2 well-being (though not depression and anxiety).

### Differences between GORD and silent reflux

As can be seen from Table 3, there were no significant differences between parents of infants with standard GORD and those with silent reflux on baseline (Time 1) measures of anxiety, well-being, illness appraisals, illness uncertainty, self-compassion, feeding satisfaction, sleep quality, social support, relationship satisfaction, time taken from symptoms to diagnosis, and management of symptoms. A significant difference between the standard GORD and silent reflux groups was found on the measure of depression (PHQ-8). However, when the Bonferroni correction for multiple comparisons was applied, this was no longer significant, suggesting no robust differences existed between the two groups.

**Table 3.** A comparison of parents of infants with GORD and those with silent reflux, from the Time 1 data.

Measure (from time 1)	Standard GORD (*n* = 142) Mean (SD)	Silent reflux (*n* = 147) Mean (SD)	*t* (287)
GAD-7	12.75 (5.594)	12.29 (6.312)	.646
Well-being	18.476 (3.202)	18.746 (3.369)	−.696
PHQ-8	12.83 (5.781)	11.22 (6.279)	2.270*^,a^
Illness appraisals	50.12 (12.111)	48.99 (11.840)	.804
Illness uncertainty	98.48 (19.617)	102.57 (17.539)	−1.871
Self-compassion	2.41 (0.723)	2.52 (0.708)	−1.336
Feeding satisfaction	5.34 (2.689)	5.56 (2.938)	−.663
Sleep quality	6.60 (2.236)	6.67 (2.162)	−.262
Social support	5.06 (2.570)	5.00 (2.593)	.185
Relationship satisfaction	5.81 (2.443)	5.95 (2.582)	−.459
Time taken from symptoms to diagnosis	0.96 (1.368)	1.04 (1.384)	−.513
Management of symptoms	57.87 (27.391)	54.63 (27.572)	1.002

*p < .05.

^a^This finding did not remain significant when Bonferroni corrected for multiple comparisons.

## Discussion

This study examined the prevalence and predictors of mental health difficulties in parents of infants (<1 year old) with GORD, using an online survey conducted over two time-points separated by 8 weeks. The results suggest that the participants experienced high rates of anxiety and depression, with an estimated 66.0% experiencing generalised anxiety disorder and 63.4% suffering from a depressive disorder, at baseline. These levels are elevated compared to both the general population (Kroenke et al., 2009; Lowe et al., 2008) and a general perinatal sample (Heron et al., 2004). This is consistent with Reflux Infants Support Association (2017) and suggests that infant GORD is a risk factor for poorer parental mental health. It is also consistent with a wealth of child illness literature that demonstrates that carers of children with a chronic illness experience significantly higher stress levels and poorer well-being than caregivers of healthy children (Cousino & Hazen, 2013).

While elevated levels of anxiety and depression were observed at both time-points, it was encouraging that there was a significant improvement in anxiety and depression scores over time. Well-being, feeding and sleep satisfaction, and parents’ perceptions of how well their infant’s GORD was managed also significantly improved between time-points. This is consistent with current guidelines suggesting that infant GORD symptoms usually become less severe over time (National Institute for Health and Care Excellence, 2015). Alternatively, the reduction in anxiety and depression levels over time could be due to regression to the mean.

The study also sought to examine whether there were psychological predictors of parents’ mental health. Baseline illness appraisals, illness uncertainty and self-compassion all independently predicted depression, anxiety and well-being scores at baseline and at 8-week follow-up, including when the control variables were added. When all predictor (and control) variables were included together in the same regression model, only self-compassion and illness uncertainty remained significant predictors of outcomes at baseline, and only self-compassion remained a significant predictor of all outcomes at follow-up.

The endurance of self-compassion as a significant predictor of better outcomes is in line with previous research that has consistently linked a person’s ability to be self-compassionate in the face of difficulties with better psychological health (Neff, 2003; Neff et al., 2007). This may be because self-compassion appears to support greater emotional self-regulation through a range of adaptive coping strategies, such as reduced defensiveness, self-blame and rumination (Terry & Leary, 2011). While the correlational nature of the current study precludes definitive causal conclusions, when its findings are taken together with this previous literature on self-compassion, they suggest that self-compassion could be a worthwhile, proximal, intervention target in future intervention research with parents with infants with GORD. Moreover, it is already known that self-compassion can be successfully improved by psychological interventions (Kirby et al., 2017).

Illness uncertainty was another significant predictor of poorer outcomes at baseline, but not follow-up, when included in the model with all predictor and control variables. Given that times of transition are often periods when illness uncertainty is heightened (Kerr & Haas, 2014), these findings might be explained by illness uncertainty playing a negative role when parents are experiencing the transition to living with their infant’s GORD (and also, for some, the transition into new parenthood), but it having a diminishing effect as these become more familiar and better-known experiences. The finding that the predictive effect of illness uncertainty diminishes over time suggests that, in contrast to self-compassion, it is not a strong candidate to be a proximal target for intervention. Similarly, illness appraisals were not consistent predictors of outcomes, at least when the other predictors were also included, and so do not appear to be promising intervention targets.

Turning to silent reflux, the hypothesis that parents whose infants suffer from this would have higher illness uncertainty, anxiety and depression, and lower well-being, was not supported. In fact, exploratory analyses revealed no robust significant differences between the silent reflux and standard GORD groups on any of the variables, despite the relatively large sample size. This draws into question the anecdotal evidence that parents whose infants have silent reflux experience greater uncertainty due to greater difficulty in diagnosing the condition compared to standard GORD (cf. Blanch & Reflux Infants Support Association, 2010). Contrary to this, in the current study, the time taken to diagnosis did not significantly differ between the silent reflux and standard GORD groups. As this is the first known study to quantitively explore differences in parental experience between these two groups, further research should test the replicability of these findings.

### Limitations

The study has the following limitations. First, the sample comprised primarily UK-based Caucasian mothers who were well-educated and either married or co-habiting, limiting the generalisability of the findings to other parents. Second, other forms of selection bias may have occurred; for example, it is possible that parents' mental health may have influenced their likelihood of participating, biasing the prevalence statistics. Third, the correlational nature of the design prohibits causal conclusions. Fourth, the use of self-report questionnaires for all measures means that shared method variance may have inflated the observed associations (cf. Podsakoff et al., 2012). Fifth, there was substantial participant attrition between the two timepoints, potentially introducing further selection bias (though Little’s MCAR test provided no evidence for this). Sixth, although the choice of control variables was based on existing literature, there were likely unmeasured variables that confounded the findings. Seventh, whether or not participants were receiving mental health treatment or support was not measured, meaning that this potentially relevant predictor/confounder could not be included in the analyses. Eighth, whilst some variables significantly predicted outcomes, a substantial amount of outcome variance in remained unaccounted for.

### Conclusions

Parents of infants with GORD showed high rates of anxiety and depression, which were elevated compared to those that have been found in perinatal and general population samples. Self-compassion was a consistent predictor of lower parental depression and anxiety and higher well-being, both cross-sectionally and 8 weeks later. The findings regarding illness uncertainty and illness appraisals were more mixed. Therefore, this first study to explore predictors of parental mental health in the context of infant GORD suggests that, of the measured predictors, self-compassion has the most promise as a proximal intervention target. Future research could benefit from examining the efficacy of a compassion-focussed intervention in this population.

## Supplemental Material

Supplemental material - Paediatric gastroesophageal reflux disease and parental mental health: Prevalence and predictors Click here for additional data file. Supplemental material for Paediatric gastroesophageal reflux disease and parental mental health: Prevalence and predictors by Elizabeth G. M. Aizlewood, Fergal W. Jones, and Rachel Whatmough in Clinical Child Psychology and Psychiatry.
