# Supplementary material for: Paediatric gastroesophageal reflux disease and parental mental health: Prevalence and predictors
Source: Clin Child Psychol Psychiatry. 2023 Mar 20;28(3):1024–37. doi: 10.1177/13591045231164866 (PMC10280658; doi:10.1177/13591045231164866)
Supplement: Supplemental material - Paediatric gastroesophageal reflux disease and parental mental health: Prevalence and predictors [file sj-pdf-1-ccp-10.1177_13591045231164866.pdf]

Online Supplementary Material

Figure OS-1

Participant Flow Diagram. Note that participants with incomplete baseline measures remained eligible to participate in the follow-up survey.

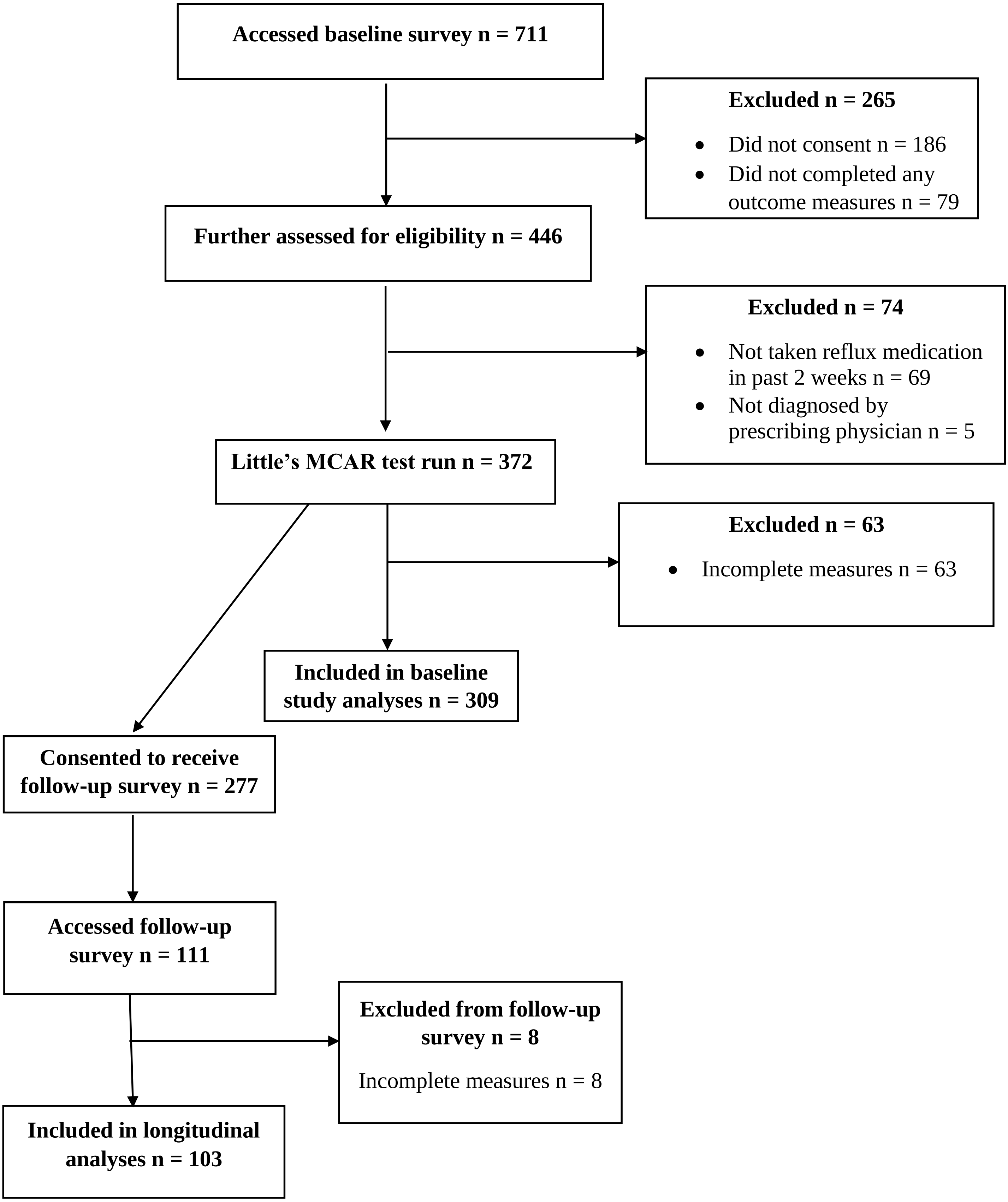

Figure OS-2

GAD-7 Total Scores at Baseline and Follow-up

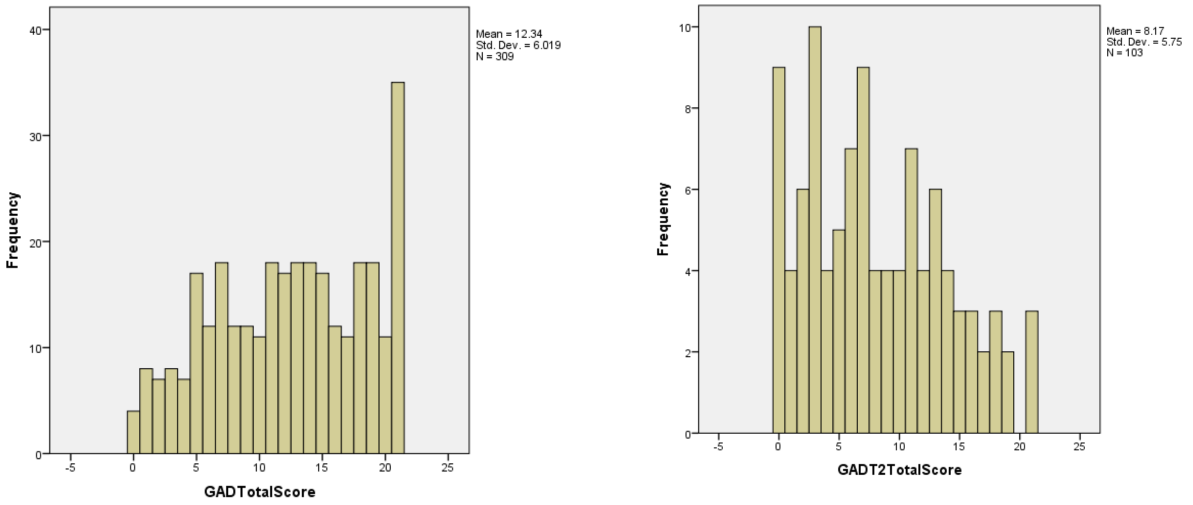

Figure OS-3

PHQ-8 Total Scores at Baseline and Follow-up.

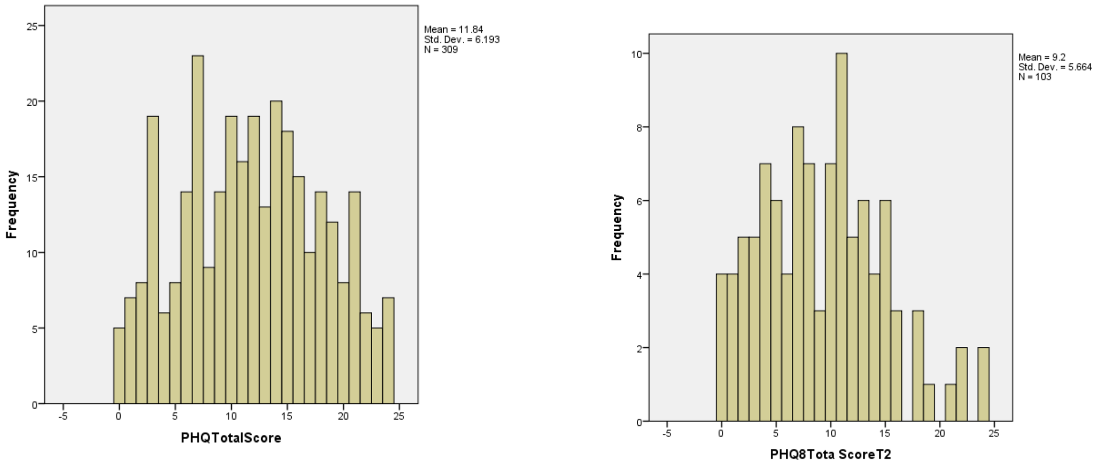

Table OS-1
Descriptive Statistics and Paired Sample t -Tests

**Measure** (Range)	**Mean Baseline** (SD) N=309	**Mean Follow-up** (SD) N=103	***t*** (102)
GAD-7 (0-21)	12.34 (6.02)	8.17 (5.75)	6.881***
SWEMWBS (0-35)	18.79 (3.62)	20.57 (4.29)	-4.674***
PHQ-8 (0-24)	11.84 (6.19)	9.20 (5.66)	4.455***
B-IPQ (0-80)	49.33 (11.93)	-	-
PPUS (35-155)	100.36 (18.57)	-	-
SCS-SF (0-5)	2.49 (0.72)	-	-
Sleep (0-10)	6.61 (2.18)	6.10 (2.06)	2.046*
Social Support (0-10)	5.04 (2.61)	5.25 (2.22)	-1.112
Relationship Support (0-10)	5.90 (2.52)	6.28 (2.42)	-.510
Feeding Satisfaction (0-10)	5.53 (2.80)	6.72 (2.33)	-3.300**
Symptom management (0-100)	56.93 (27.23)	70.73 (23.42)	-5.091***

***p<0.001; **p<0.01; *p<0.05

Table OS-2

Participants Scoring Above Clinical Cut-Off on Measures of Anxiety (GAD-7) and Depression (PHQ-8)

**Measure**	**Number above clinical cut off (≥10) indicating mental health difficulties.**			
	**Initial Survey Sample** (N= 309)	**95% Confidence Interval for Proportion Above Cut-Off**	**Follow-up Sample** (N=103)	**95% Confidence Interval for Proportion Above Cut-Off**
	**N (%)**		**N (%)**	
GAD-7 (Anxiety)	204 (66.0%)	60.4-71.3%	41 (39.8%)	30.3-49.9%
PHQ-8 (Depression)	196 (63.4%)	57.8-68.8%	50 (48.5%)	38.6-58.6%

GAD-7 = Generalized Anxiety Disorder Screener 7; PHQ-8 = Patient Health Questionnaire 8

Table OS-3

Simple linear regressions for each predictor and outcome combination, with each row representing a separate regression analysis.

Outcome variable	Predictor (at baseline)	R^2	F(df)	Coefficient (Beta)	*t*-value
GAD-7 Baseline	Illness Appraisals	.171	63.220*** (1, 307)	.208	7.951***
	Illness Uncertainty	.179	67.071*** (1, 307)	.137	8.190***
	Self-compassion	.178	66.573*** (1, 307)	-3.510	-8.159***
	Social support	.052	16.889*** (1, 307)	-.527	-4.110***
	Relationship satisfaction	.120	41.763*** (1, 307)	-.826	-6.462***
	Sleep quality	.055	17.979*** (1, 307)	.649	4.240***
	Feeding satisfaction	.127	44.478*** (1, 307)	-.764	-6.669***
GAD-7 Follow-up	Illness Appraisals	.224	29.206*** (1, 101)	.216	5.404***
	Illness Uncertainty	.173	21.154*** (1, 101)	.124	4.599***
	Self-compassion	.258	35.190*** (1, 101)	-3.950	-5.932***
	Social support	.115	13.140*** (1, 101)	-.827	-3.625***
	Relationship satisfaction	.074	8.019** (1, 101)	-.661	-2.832**

	Sleep quality	.019	1. 914 (1, 101)	.357	1.383
	Feeding satisfaction	.107	12.097** (1, 101)	-.748	-3.478**
PHQ-8 Baseline	Illness Appraisals	.139	49.634*** (1, 307)	.194	7.045***
	Illness Uncertainty	.144	51.491*** (1, 307)	.126	7.176***
	Self-compassion	.229	90.99*** (1, 307)	-4.090	-9.539***
	Social support	.070	23.283*** (1, 307)	-.631	-4.825***
	Relationship satisfaction	.152	55.085*** (1, 307)	-.958	-7.422***
	Sleep quality	.102	34.945*** (1, 307)	.908	5.911***
	Feeding satisfaction	.079	26.357*** (1, 307)	-.621	-5.134***
PHQ-8 Follow-up	Illness Appraisals	.186	23.107*** (1, 101)	.194	4.807***
	Illness Uncertainty	.149	17.671*** (1, 101)	.113	4.204***
	Self-compassion	.208	26.562*** (1, 101)	-3.493	-5.154***
	Social support	.059	6.305* (1, 101)	-.582	-2.511*
	Relationship satisfaction	.084	10.318** (1, 101)	-.732	-3.212**
	Sleep quality	.113	12.902** (1, 101)	.867	3.592**
	Feeding satisfaction	.054	5.777* (1, 101)	-.542	-2.404*

Well-being Baseline	Illness Appraisals	.204	78.886*** (1, 307)	-.137	-8.882***
	Illness Uncertainty	.223	88.104*** (1, 307)	-.092	-9.386***
	Self-compassion	.239	96.48*** (1, 307)	2.446	9.822***
	Social support	.095	32.281*** (1, 307)	.429	5.682***
	Relationship satisfaction	.206	79.853*** (1, 307)	.653	8.936***
	Sleep quality	.076	25.135*** (1, 307)	-.457	-5.013***
	Feeding satisfaction	.080	26.521*** (1, 307)	.364	5.150***
Well-being Follow-up	Illness Appraisals	.243	33.809*** (1, 101)	-.170	-5.815***
	Illness Uncertainty	.152	18.116*** (1, 101)	-.087	-4.256***
	Self-compassion	.191	23.778*** (1, 101)	2.531	4.876***
	Social support	.053	5.681* (1, 101)	.420	2.383*
	Relationship satisfaction	.105	11.797** (1, 101)	.588	3.435**
	Sleep quality	.035	3.694 (1, 101)	-.367	-1.922
	Feeding satisfaction	.059	6.319* (1, 101)	.414	2.514*

***p<0.001; **p<0.01; *p<0.05; GAD-7 = Generalized Anxiety Disorder Screener 7; PHQ-8 = Patient Health Questionnaire 8

Table OS-4

Multiple linear regressions at baseline with all three predictors (plus all control variables) entered as predictors into the same model. The R^2 change and F change values refer to change from a model that only included the control variables as predictors (i.e. the respective Model 1 in Table 2 in the journal article). Separate regressions were run for each outcome.

**Outcome Variable** (baseline)	**Predictors** (baseline)	**Unstandard-ized B**	***t*(301)**	**R^2**	**F (df)**	**Change from the respective Model 1**	
						**R^2 change**	**F (df) change**
GAD -7 Model 2	Social Support	.088	.673	.355	23.624*** (7,301)	.132	20.489*** (3, 301)
	Relationship	-.370	-2.642**				
	Sleep	.292	2.193*				
	Feeding	-.418	-3.671***				
	**Illness Appraisals**	**.014**	**.374**				
	**Illness Uncertainty**	**.066**	**2.952****				
	**Self-Compassion**	**-2.317**	**-5.512*****				
Well-being Model 2	Social Support	-.006	-.084	.448	34.887*** (7, 301)	.175	31.875*** (3, 301)
	Relationship	.344	4.420***				
	Sleep	-.217	-2.927**				
	Feeding	.085	1.348				
	**Illness Appraisals**	-.014	**-.659**				
	**Illness Uncertainty**	-.048	**-3.869*****				
	**Self-Compassion**	1.519	**6.494*****				
PHQ-8 Model 2	Social Support	.004	.033	.387	27.180*** (7, 301)	.143	23.401*** (3, 301)
	Relationship	-.426	-3.038**				
	Sleep	.568	4.260***				
	Feeding	-.255	-2.238*				

**Illness Appraisals**	**.000**	**-.011**
**Illness Uncertainty**	**.055**	**2.450***
**Self-Compassion**	**-2.886**	**-6.848*****

***p<0.001; **p<0.01; *p<0.05; GAD-7 = Generalized Anxiety Disorder Screener 7; PHQ-8 = Patient Health Questionnaire 8

Table OS-5

Multiple linear regressions with predictors measured at baseline (Time 1) and outcomes at follow-up (Time 2).

**Outcome Variable Model** (follow-up)	**Predictors** (baseline)	**Unstand-ardized B**	**t-Value** (df)	**R^2**	**F** (df)	**Change from the respective Model 1**	
						**R^2 change**	**F (df) change**
GAD-7 Model 1	Social Support	-.659	-2.720** (98)	.215	6.711** (4, 98)		
	Relationship	-.223	-.854 (98)				
	Sleep	.147	.579 (98)				
	Feeding	-.635	-3.052** (98)				
GAD-7 Model 2a	Social Support	-.526	-2.218* (97)	.282	7.629*** (5, 97)	**.067**	**9.089**** (1, 97)
	Relationship	-.096	-.376 (97)				
	Sleep	.010	.039 (97)				
	Feeding	-.286	-1.239 (97)				
	**Appraisals**	.151	**3.015**** (97)				
GAD-7 Model 2b	Social Support	-.549	-2.305* (97)	.270	7.191*** (5, 97)	**.055**	**7.370**** (1, 97)
	Relationship	-.195	-.771 (97)				
	Sleep	.122	.493 (97)				
	Feeding	-.374	-1.676 (97)				
	**Uncertainty**	.081	**2.715**** (97)				
GAD-7 Model 2c	Social Support	-.518	-2.360* (97)	.373	11.549*** (5, 97)	**.158**	**24.475***** (1, 97)
	Relationship	-.095	-.404 (97)				
	Sleep	-.022	-.097 (97)				
	Feeding	-.543	-2.890* (97)				
	**Self-Compass.**	-3.272	**-4.947***** (97)				
Well-being Model 1	Social Support	.217	1.156 (98)	.157	4.550** (4, 98)		

	Relationship	.387	1.917 (98)				
	Sleep	-.160	-.812 (98)				
	Feeding	.316	1.966 (98)				
Well-being Model 2a	Social Support	.084	.472 (97)	.276	7.042*** (5, 97)	**.120**	**16.017***** (1, 97)
	Relationship	.261	1.366 (97)				
	Sleep	-.023	-.123 (97)				
	Feeding	-.031	-.177 (97)				
	**Appraisals**	-.150	**-4.002***** (97)				
Well-being Model 2b	Social Support	.125	.684 (97)	.226	5.649***	**.069**	**8.626**** (1, 97)
	Relationship	.364	1.870 (97)				
	Sleep	-.138	-.730 (97)				
	Feeding	.099	.579 (97)				
	**Uncertainty**	-.067	**-2.937**** (97)				
Well-being Model 2c	Social Support	.129	.728 (97)	.266	7.033*** (5, 97)	**.109**	**14.463***** (1, 97)
	Relationship	.308	1.617 (97)				
	Sleep	-.054	-.292 (97)				
	Feeding	.259	1.708 (97)				
	**Self-Compass.**	2.030	**3.803***** (97)				
PHQ-8 Model 1	Social Support	-.401	-1.665 (98)	.202	6.219*** (4, 98)		
	Relationship	-.261	-1.006 (98)				
	Sleep	.692	2.743** (98)				
	Feeding	-.373	-1.806 (98)				
PHQ-8 Model 2a	Social Support	-.282	-1.187 (97)	.257	6.722*** (5, 97)	**.055**	**7.166**** (1, 97)
	Relationship	-.148	-.579 (97)				
	Sleep	.570	2.289* (97)				

	Feeding	-.063	-.271 (97)				
	**Appraisals**	.135	**2.677**** (97)				
PHQ-8 Model 2b	Social Support	-.283	-1.205 (97)	.268	7.086*** (5, 97)	**.065**	**8.619**** (1, 97)
	Relationship	-.232	-.926 (97)				
	Sleep	.665	2.734** (97)				
	Feeding	-.095	-.430 (97)				
	**Uncertainty**	.086	**2.936**** (97)				
PHQ-8 Model 2c	Social Support	-.285	-1.259 (97)	.311	8.753*** (5, 97)	**.108**	**15.268***** (1, 97)
	Relationship	-.157	-.644 (97)				
	Sleep	.554	2.324* (97)				
	Feeding	-.298	-1.536 (97)				
	**Self-Compass.**	-2.669	**-3.907***** (97)				

***p<0.001; **p<0.01; *p<0.05; GAD-7 = Generalized Anxiety Disorder Screener 7; PHQ-8 = Patient Health Questionnaire 8; Relationship = Relationship Satisfaction; Sleep = Sleep Quality; Feeding = Feeding Satisfaction; Self-Compass. = Self-Compassion; Appraisals = Illness Appraisals; Uncertainty = Illness Uncertainty.

Table OS-6

Multiple linear regressions with predictors measured at Time 1 and outcomes at Time 2, and all three predictors (plus all control variables) entered as predictors into the same model. The R^2 change and F change values refer to change from a model that only included the control variables as predictors (i.e. the respective Model 1 in Table OS-5). Separate regressions were run for each outcome.

**Outcome Variable** (follow-up)	**Predictors** (baseline)	**Unstand-ardized B**	***t*(95)**	**R^2**	**F** (df)	**Change from the respective Model 1** R^2 change	**F (df) change**
GAD -7 Follow-up Model 2	Social Support	-.430	-1.965	.408	9.357*** (7, 95)	.193	10.330*** (3, 95)
	Relationship	-.041	-.175				
	Sleep	-.076	-.328				
	Feeding	-.293	-1.373				
	**Appraisals**	**.068**	**1.084**				
	**Uncertainty**	**.032**	**.881**				
	**Self-Compass.**	**-2.915**	**-4.367*** **				
Well-being Follow-up Model 2	Social Support	.033	.192	.344	7.124*** (7, 95)	.188	9.059*** (3, 95)
	Relationship	.226	1.223				
	Sleep	.030	.164				
	Feeding	-.023	-.136				
	**Appraisals**	-.112	**-2.277***				
	**Uncertainty**	-.011	**-.383**				
	**Self-Compass.**	1.624	**3.099****				
PHQ-8 Follow-up Model 2	Social Support	-.195	-.866	.352	7.384*** (7, 95)	.150	7.331*** (3, 95)
	Relationship	-.123	-.506				
	Sleep	.523	2.199*				
	Feeding	-.050	-.228				
	**Appraisals**	**.035**	**.538**				

**Uncertainty**	**.055**	**1.468**
**Self-Compass.**	**-2.319**	**-3.371****

***p<0.001; **p<0.01; *p<0.05; GAD-7 = Generalized Anxiety Disorder Screener 7; PHQ-8 = Patient Health Questionnaire 8; Relationship = Relationship Satisfaction; Sleep = Sleep Quality; Feeding = Feeding Satisfaction; Self-Compass. = Self-Compassion; Appraisals = Illness Appraisals; Uncertainty = Illness Uncertainty.
